# The interaction of TEA domain transcription factor 4 (TEAD4) and Yes-associated protein 1 (YAP1) promoted the malignant process mediated by serum/glucocorticoid regulated kinase 1 (SGK1)

**DOI:** 10.1080/21655979.2021.1882142

**Published:** 2021-02-10

**Authors:** Songlin He, Hanlu Zhang, Zongwei Xiao, Sandeep Bhushan, Ke Gao, Wenping Wang

**Affiliations:** a Department of Cardiothoracic Surgery, The Second People’s Hospital of Chengdu, Chengdu, Sichuan, China; b Department of Thoracic Surgery, West China Hospital of Sichuan University, Chengdu, Sichuan, China

**Keywords:** TEAD4, yap1, sgk1, esophageal squamous cell carcinoma

## Abstract

TEA domain transcription factor 4 (TEAD4) has been investigated to be implicated in the progression of various cancers, and it plays a role in the esophageal squamous cell carcinoma (ESCC). The study was designed to investigate how TEAD4 affected the progression of ESCC through Hippo signaling pathway *in vitro* and *in vivo*. The interaction of TEAD4 and Yes-associated protein (YAP) was detected though immunoprecipitation assay (IP). Following the treatment of TED-347, which was able to suppress the interaction of TEAD4 and YAP1, the malignant behaviors of cells including proliferation, invasion, and migration were assessed by EDU staining, wound healing, and transwell assay *in vitro*, while tumor growth was measured. Luciferase reporter plasmids containing the enhancer and promoter region of serum/glucocorticoid regulated kinase 1 (SGK1) were constructed to analyze how TEAD4 affected the transcription of SGK1. The above cell behaviors were further analyzed after the silencing of SGK1. Results showed that TED-347 hindered the promoting effect of TEAD4 overexpression on the malignant behaviors of ESCC cells, and this effect was related to the suppression of the TEAD4/YAP1 complex. Moreover, the promoter activity of SGK1 was obviously inhibited by TED-347. Decreased expression of SGK1 suppressed the above behaviors of cells and destroyed the effects of increased expression of TEAD4. Collectively, TEAD4/YAP promotes the malignant process of ESCC cells, which was inhibited by the interference of SGK1. Targeting TEAD4/YAP1 complex or SGK1 could find application in the treatment of esophageal squamous cell carcinoma.

## Graphical Abstract

**Figure:**
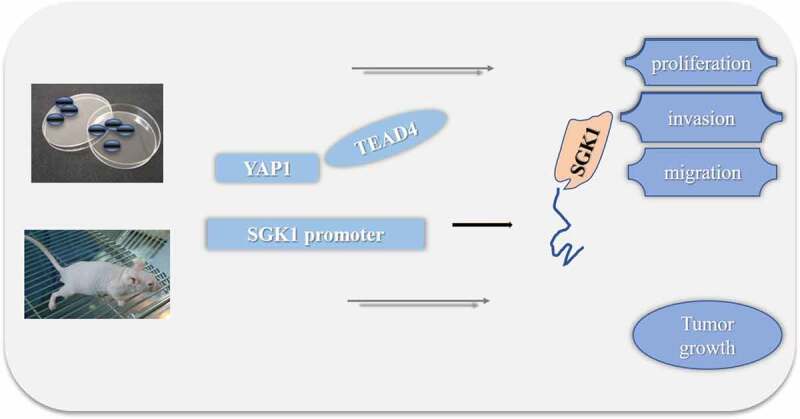

## Introduction

Esophageal cancer is one of the least known and deadliest cancers in the world, mainly because of its aggressive nature and low survival rate [1]. Since most patients with esophageal cancer were diagnosed as locally advanced or had distant metastases upon hospitalization, the 5-year survival rate of patients with esophageal cancer was 15–25% [2].

YAP and the transcriptional co-activator with PDZ-binding motif (TAZ) transcriptional coactivator, which are key effectors of the Hippo signaling pathway, can enter the cell nucleus to bind to TEAD4 or other transcription factors, thereby inducing the proliferation process of cells [3]. In recent years, the effects of TEAD4 on promoting cancer progression have been gradually concerned. TEAD4 can form a transcription complex with YAP or independently regulate the expression of related downstream target genes independent of YAP, and play an oncogenic role in gastrointestinal tumors, leading to the occurrence and progression of tumors [4–8].

Our previous study found that TEAD4 interference could inhibit the proliferation, invasion, and migration of ESCC cells. Therefore, the present study further investigated how Hippo signally pathway proteins that are implicated in the malignant behaviors of ESCC cells were affected by TEAD4. Serum and glucocorticoid-regulate protein kinase 1 (SGK1), a serine/threonine protein kinase, could form a positive feedback loop with TEAD-YAP/TAZ complex [9]. It was identified as a potential molecular target for the treatment of endometrial cancer, the inhibition of which caused autophagy, apoptosis, and endoplasmic reticulum stress [10]. Additionally, Sgk1 levels in patients with ESCC after neoadjuvant chemotherapy have been found to be associated with an overall worse prognosis [11]. The transcriptional upregulation of SGK1 was confirmed to be dependent on the TEAD-YAP transcription factor-coactivator complex [9]. CTGF was identified as one of the induction genes by YAP and it was involved in cell growth [12]. Based on these, we presented a hypothesis that TEAD could formed a complex with YAP, which was involved in the malignant progression of ESCC cells. This effect could be mediated by a downstream target, SGK1. Thus, the current study intended to investigate whether SGK1 could act as the downstream target of the TEAD-YAP complex to involve in the progression of esophageal squamous cell carcinoma.

## Methods

Cell line

Human ESCC cell line, KYSE-30, was purchased from ATCC (USA) and cultured in RPMI 1640 medium containing 10% fetal bovine serum, 100 U/mL penicillin, and 100 mg/L streptomycin at 37°C with 5% CO_2_.

Plasmid transfection

KYSE-30 cells were seeded into a 6-well plate (4 × 10^5^ cells) and cultured at 37°C with 5% CO_2_. 2 mL DMEM medium containing 10% FBS was added to each well. When the cell density reached 60% ~ 70%, cells were transfected with OV-TEAD4, OV-NC, ShRNA – SGK1 or ShRNA-NC lentivirus for 12 h (0.5 ml each, HANBIO, Shanghai, China). The original medium was discarded and 2 mL of DMEM containing 10% FBS, 10^5^ U· L^−1^ penicillin, and 100 g· L^−1^ streptomycin was added to each well. The culture was continued in the incubator for 36 h. The cells were collected for further study.

RT-qPCR

KYSE-30 cells were collected and total RNA was extracted using the Trizol method (Tokara, Japan). The purity and concentration of total RNA were determined by the Ultraviolet Spectrophotometry. cDNA was obtained by reverse transcription according to the instructions of the reverse transcription reaction kit (Tokara, Japan), and PCR was performed using cDNA as the template.

Western blot

KYSE30 was collected 48 h after transfection in each group. The total protein of each group was extracted with RIPA lysis solution (Thermo Fisher Scientific). About 80 μg protein sample of each group was performed and separated using SDS-PAGE. The proteins were transferred to polyvinylidene fluoride (PVDF) membranes at low temperature. Proteins were locked using the blocking solution at room temperature for 60 min and then incubated at 4°C overnight with primary antibodies. After being washed by PBS, the secondary antibody labeled with Horseradish peroxidase (HRP) was added and incubated with protein bands at room temperature for 50 min. Electrochemiluminescence (ECL) method was used to develop color. The relative levels of target proteins relative to GADPH were calculated.

Immunoprecipitation assay (IP)

The collected cells were lysed on ice for 30 min and then centrifuged at 14,000 × g at 4°C for 15 min. A part of the collected supernatant was used to perform Western blotting analysis of YAP1 and TEAD4 proteins. Anti-TEAD4 antibody (Abcam, England) was added into other part of the supernatant for incubation at 4°C overnight. Next, Protein A/G agarose was also added for incubation for 2 h. Then, the supernatant was discarded after the above mixture was centrifuged at 14,000 × g at 4°C for 15 s. SDS-loading buffer (15 µL) was then added to the immunoprecipitation, which then was boiled for 5 min and used for Western blotting assay.

CCK8 assay

KYSE30 cells in each group were seeded into 96-well plates and cultured at 37°C, respectively. After being cultured for 24, 48, and 72 h, respectively, 10 μL CCK8 solution was added to the wells. The optical density (OD) at 450 nm was detected by an enzyme micrometer after 2 h of incubation.

Colony formation assay

KYSE30 cells were digested and resuspended in a DMEM medium containing 10% FBS. One thousand cells per well were inoculated into six-well plates with three multiple wells in each group. 2 mL DMEM containing 10% FBS was added and incubated in an incubator with 5% CO_2_ for 2 ~ 4 weeks, when the clonal morphology was observed dynamically. When visible clones appeared, the culture was stopped. After PBS washing for three times, 4% paraformaldehyde was added for fixation for 15 min. The cells were stained with 5 mL crystal violet for 30 min. After being washed with PBS for three times, the cells were observed under a 10-fold microscope. The isolated cell population with a cell number ≥10 was taken as a clone.

EDU staining

KYSE30 cells were seeded into a 96-well plate. After indicated treatment, 100 µL EdU solution was added to cells (Abcam, England). After 2 h, EdU solution was discarded and cells were washed by PBS twice. Then, cells were fixed using methanol solution. Next, the penetrating agent was used to incubate cells for 15 min. Subsequently, cells were incubated with Apollo staining solution for 10 min, followed by incubation by DAPI addition (100 µL) at room temperature for 30 min. The staining result was observed under a fluorescence microscope.

Wound healing assay

KYSE30 cells in each group were seeded in six-well plates at a concentration of 5*10^5^/well. The scratch was drawn evenly with a 100 μL pipette tip. After washing with PBS, a serum-free DMEM medium was added. After 48 h, the scratch was photographed under a fluorescence microscope.

Transwell assay

Matrigel was diluted with a serum-free medium at a ratio of 1:8. About 55 μL Matrigel was added to the bottom of the chamber. After 1 h, the non-solidifying medium was removed. DMEM without FBS was used to adjust the cell concentration to 5*10^8^/L. About 200 μL cell suspension containing 10% FBS was added into the upper compartment. In the lower chamber, 600 μL DMEM containing 10% FBS was added and incubated at 37°C with 5% CO_2_ for 48 h. After that, the cells were taken out, the culture medium was discarded, and the unpassed cells were carefully wiped off with a cotton swab. The cells were washed with PBS for three times, and then fixed using 4% paraformaldehyde for 30 min. One percent crystal violet 600 μL was used to stain cells for 30 min. The cells that were observed in the field were the invading cells. Five fields were randomly selected to count the number of invading cells.

Animal experiment

Healthy adult male BALB/c nude mice (N = 20, 6 weeks) were purchased from Beijing Zhishan Co., Ltd (Beijing, China), weighing 20 ± 2 g (with four in each group). The mice were fed with a normal diet and free access to water. In the first part of the experiment, the KYSE30 cells with TEAD4 overexpression or TEAD4 overexpression combined with TED347 treatment were injected subcutaneously into the nude mice. In the second part of the experiment, the KYSE30 cells overexpressing TEAD4 or those co-transfected with TEAD4 overexpression lentivirus and SGK1 silencing lentivirus were injected subcutaneously into the nude mice. Starting from the 3rd day, the body weight and tumor volume were weighed every 3 days. After the weight and tumor volume were measured on the 21th day, the nude mice were anesthetized with ethane and sacrificed using the cervical dislocation. Tumor tissue was taken out and photographed. The study was approved by the ethics committee of West China Hospital of Sichuan University.

Immunohistochemical assay

Paraffin section of tumor tissue was prepared and then dewaxed for 10 min twice. Then, sections were rehydrated with gradient alcohol, 100%, 95%, 85%, 75% alcohol for 5 min in sequence. After being rinsed with PBS for 5 min, the sections were incubated with 3% H_2_O_2_ at room temperature in the dark for 20 min. Each section was dripped with 10% serum about 50 μL and blocked at room temperature for 30 min. The primary antibody against-Ki67 (abcam, England) was added and incubated with the sections at 4°C overnight. Afterward, the primary antibody was added and incubated with the sections for 20 min at 37°C. About 1 ml of fresh DAB working solution was prepared and added to each section (50 μl). The color development was stopped after washing by running water. Next, the sections were stained using Hematoxylin for 2 min. Finally, an appropriate amount of neutral gum was used to mount the sections.

Luciferase reporter gene assay

The SGK1 promoter region and genomic region 7807 or its mutated region on the YAP1-binding motif were cloned into the luciferase reporter gene plasmids (HANBIO, Shanghai, China). These plasmids and OV-TEAD4 or in combination with TED-347 were used to co-treat KYSE-30 cells using Transfection reagents (thermo scientific) for culture for 48 h. Then, after cells were lysed using 1XPLB, Luciferase Assay Reagent II (100 μL) was added and the intensity of luciferase reaction was detected.

Statistical analysis

Data are expressed as the mean ± standard deviation. GraphPad 8.0 statistical software (USA) was used for all statistical analyses. Comparisons among groups were analyzed by one-way analysis of variance followed by Tukey’s test. P < 0.05 was considered to indicate a statistically significant difference.

## Results

TEAD4 interacted with YAP1

**Figure 1.**
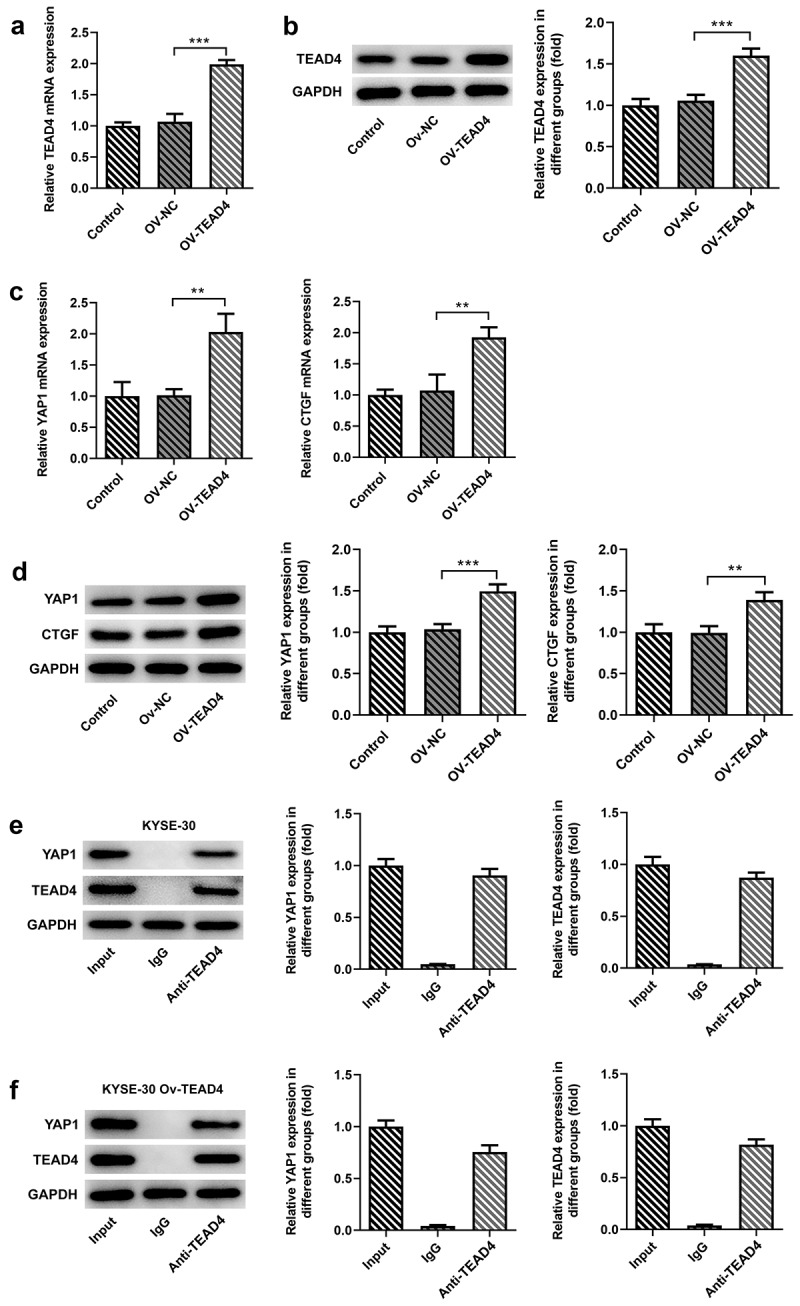
TEAD4 interacted with YAP1 in KYSE30 cells. The overexpression effects of OV-TEAD4 plasmids in KYSE30 cells were analyzed through RT-qPCR (a) and Western blot(b). the detection of YAP1 and CTGF expression through RT-qPCR (c) and Western blot (d). the analysis of immunoprecipitation pulled down by magnetic bead-TEAD4 antibody for YAP1 and TEAD4 expression in KYSE-30 cells (e) and KYSE-30 cell with OV-TEAD4 plasmids transfection (f). The experimental data were presented as mean±SD. Asterisks indicate that difference between two groups is statistically significant. **p < 0.01, ***p < 0.001

To analyze the effect of the overexpression plasmid of TEAD4 in KYSE30 cells, the transcription and translation levels of TEAD4 were, respectively, detected through RT-qPCR and Western blot assay. Higher expression of TEAD4 at mRNA and protein levels have been detected in KYSE30 cells transfected with OV-TEAD4 plasmids as compared to cells with OV-NC transfection (Figure 1a, B). TEAD has been confirmed to engage in YAP-dependent gene induction, such as CTGF [12]. Thus, we wondered whether the induction of TEAD4 overexpression affected the levels of YAP1 and CTGF. As shown in Figure 1 C, D, in comparison to the OV-NC group, a significant increase in the expression of YAP1 and CTGF was observed in the OV-TEAD4 group, demonstrating that TEAD4 overexpression promoted the expression of YAP1 and CTGF. Next, YAP1 was remarkably elevated by Western blot assay through analyzing the immunoprecipitation which was pulled down by magnetic bead-TEAD4 antibody complex in KYSE-30 cells and KYSE-30 cells overexpressing TEAD4 (Figure 1e, F), indicating that YAP1 interacted with TEAD4.

TEAD4 promoted proliferation of KYSE-30 cells by forming a complex with YAP1

**Figure 2.**
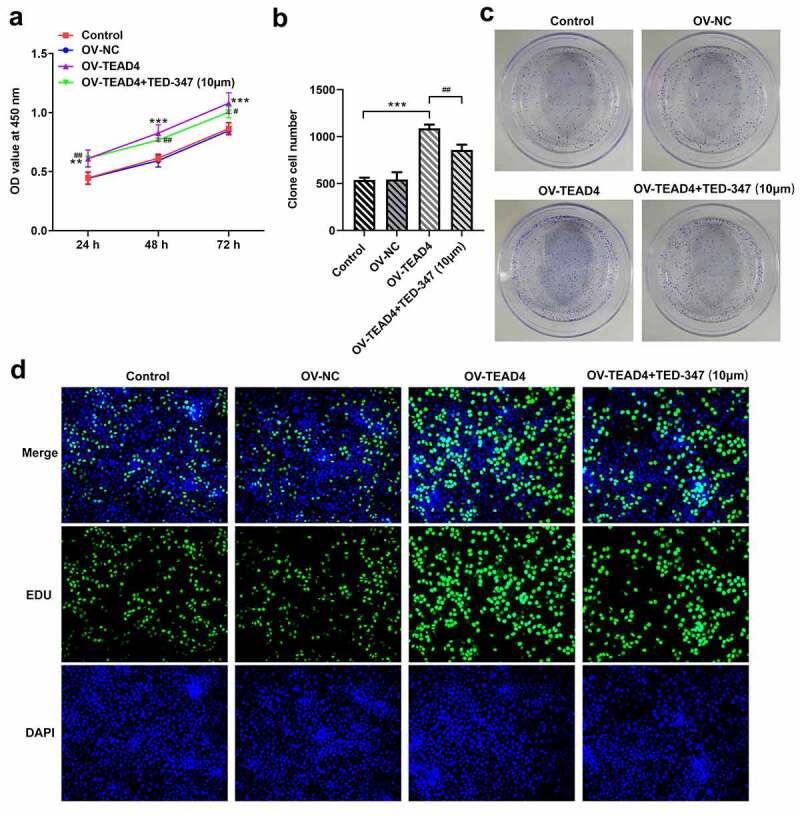
TEAD4-YAP1 complex induced proliferation of KYSE-30 cells. The detection of proliferation of KYSE-30 cells through CCK8 assay (a), colony formation (b-c) and EDU staining (d). The experimental data were shown as mean±SD. Asterisks or hashes indicate that difference between two groups is statistically significant. **p < 0.01, ***p < 0.001 versus Ov-NC, ^#^p < 0.05, ^##^p < 0.01, ^###^p < 0.001 versus OV-TEAD4

In order to test whether TEAD4 plays a role by combining with YAP1, TED-347, a covalent allosteric inhibitor of the TEAD-YAP protein–protein interaction, was used to inhibit the interaction between TEAD4 and YAP1. The proliferation of KYSE-30 cells was markedly enhanced by transfecting TEAD4 overexpression plasmids through analysis of CCK8 assay, colony formation assay, and EDU staining (Figure 2 A–D), but these effects were significantly blocked by 10 µm TED-347 addition. These results indicated that the role of TEAD in promoting cell proliferation was attributed to the formation of the TEAD4-YAP1 complex.

The interaction of TEAD4 and YAP1 facilitated the migration and invasion of KYSE-30 cells

**Figure 3.**
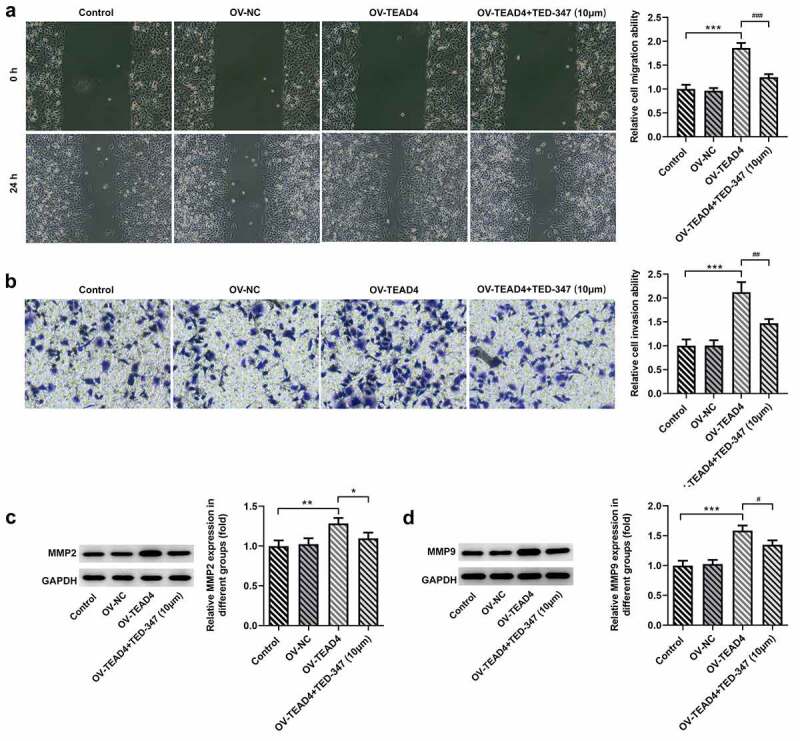
The enhancement of the migration and invasion of KYSE-30 cells through interaction of TEAD4 and YAP1. The detection of migration (a) and invasion (b) of KYSE-30 cells, as well as the assessment of expression of MMP2 and MMP9 through Western blot assay (c-d). The height of the bar chart indicated the mean (±SD) of experimental data. Asterisks or hashes indicate that difference between two groups is statistically significant. **p < 0.01, ***p < 0.001 versus Ov-NC. ^#^p < 0.05, ^##^p < 0.01 versus OV-TEAD4

In order to further analyze the possible action mechanism of TEAD4-YAP1 interaction, wound healing, Transwell assay, and Western blot were, respectively, carried out to analyze the migration and invasion of KYSE-30 cells. Our data showed that TEAD4 overexpression promoted the migration and invasion of KYSE-30 cells (Figure 3a, B). Treatment with TED-347 led to a marked decrease in the migration and invasion of these cells (Figure 3a). Additionally, a significant reduction was also observed in the expression levels of MMP2 and MMP9 after TED-347 treatment, compared to the HG group (Figure 3c, D).

The interaction of TEAD4 and YAP1 promoted tumor growth

**Figure 4.**
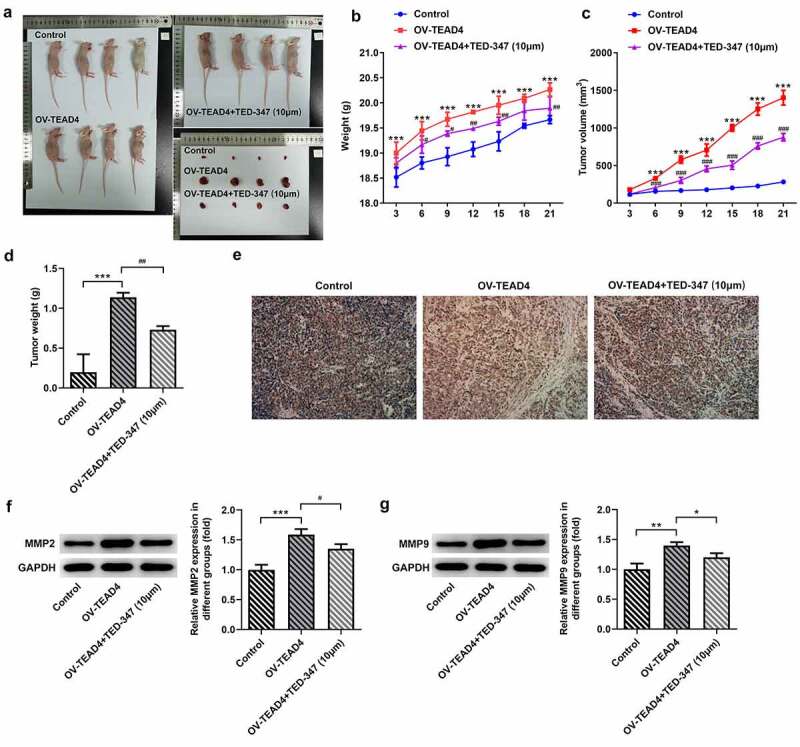
The subcutaneous injection of KYSE-30 cells with TEAD4 overexpression or in combination with TED-37 treatment affects tumor growth. After induction of 21 days, the photos of mice in different groups (a). The weight (b), tumor volume (c), and tumor weight (d) were detected. The immunohistochemical staining of Ki67 (e) and the expression levels of MMP2 and MMP9 through Western blot (f–g). The experimental data was shown as mean±SD. Asterisks or hashes indicate that difference between two groups is statistically significant. **p < 0.01, ***p < 0.001 versus control, ^#^p < 0.05, ^##^p < 0.01 versus OV-TEAD4

It could be seen in Figure 4a–D that TEAD4 overexpression could promote esophageal squamous cell carcinoma growth and increase mice weight compared with the control group. However, KYSE-30 cells with TED-347 addition could markedly block the effect of TEAD4 overexpression. Then, the tumor tissues in different groups were used to perform an immunohistochemical assay for the detection of Ki67 expression. A significant increase in positive staining of Ki67 was observed in the OV-TEAD4 group compared with the control group, while a significant reduction appeared in the cotreatment group of OV-TEAD4 and TED-347 (Figure 4e). Additionally, a similar trend caused by TEAD4 overexpression or in combination with TED-347 treatment was also found in MMP2 and MMP9 expression (figure 4f, G).

TEAD4-YAP1 complex promoted the expression of SGK1

**Figure 5.**
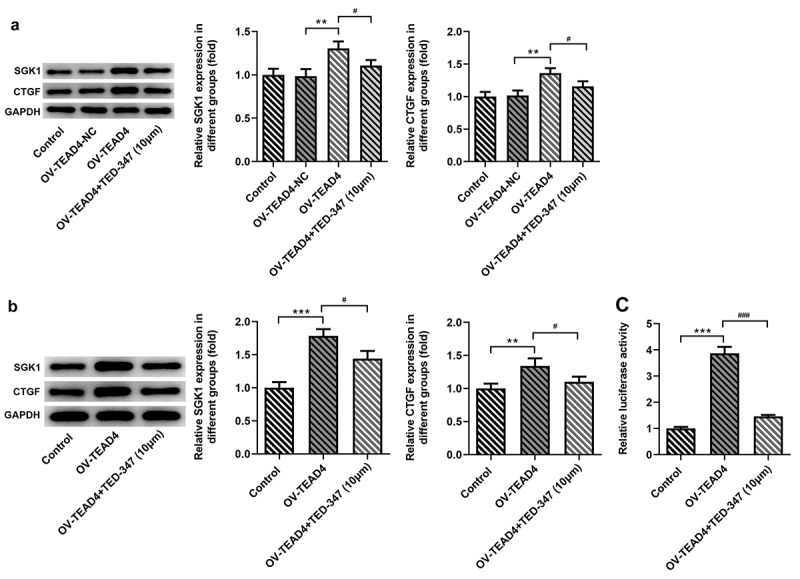
TED-347 blocked the effects of TEAD4 overexpression on the protein levels of SGK1 and CTGF. Western blot analysis of SGK1 and CTGF in KYSE-30 cells with OV-TEAD4 transfection or in combination with TED-347 treatment, **p < 0.01, ^#^p < 0.05 (a) and tumor-bearing mice (b). The protein levels were normalized to that of GAPDH. The detection of luciferase activities through the transfection of OV-TEAD4 or in combination with TED-347 treatment (c). The experimental data were represented by mean±SD. Asterisks or hashes indicate that difference between two groups is statistically significant. **p < 0.01, ***p < 0.001 versus ^#^p < 0.05 ^###^p < 0.001

From the above results, TEAD4 accelerated the deterioration of ESCC *in vivo* and *in vitro* by forming a complex with YAP1. YAP1 was reported to promote the expression of SGK1 [9], so this study continued to explore whether TEAD4 can promote the expression of SGK1 by interacting with YAP1. In *in vitro* study, the expression of SGK and CTGF was significantly increased through inducing the expression of TEAD4 in KYSE-30 cells (Figure 5a), but their expression was reduced by TED-347. Next, in *in vivo* study, by analyzing the protein levels of SGK1 and CTGF in tumor tissues of mice bearing tumor, similar effects were also observed in different groups (Figure 5b). Subsequently, luciferase reporter gene plasmids containing SGK1 promoter region and enhancer region were constructed and cotransfected into KYSE-30 cells with TEAD4 overexpression plasmids or with TEAD4 overexpression plasmids in combination with TED-347 addition. The results found that TEAD4 overexpression markedly enhanced luciferase activities, but this effect was markedly counteracted by TED-347, indicating that the interaction of TEAD4 and YAP1 enhanced the promoter activities of SGK1.

SGK1 silencing suppressed the proliferation of KYSE-30 cells

**Figure 6.**
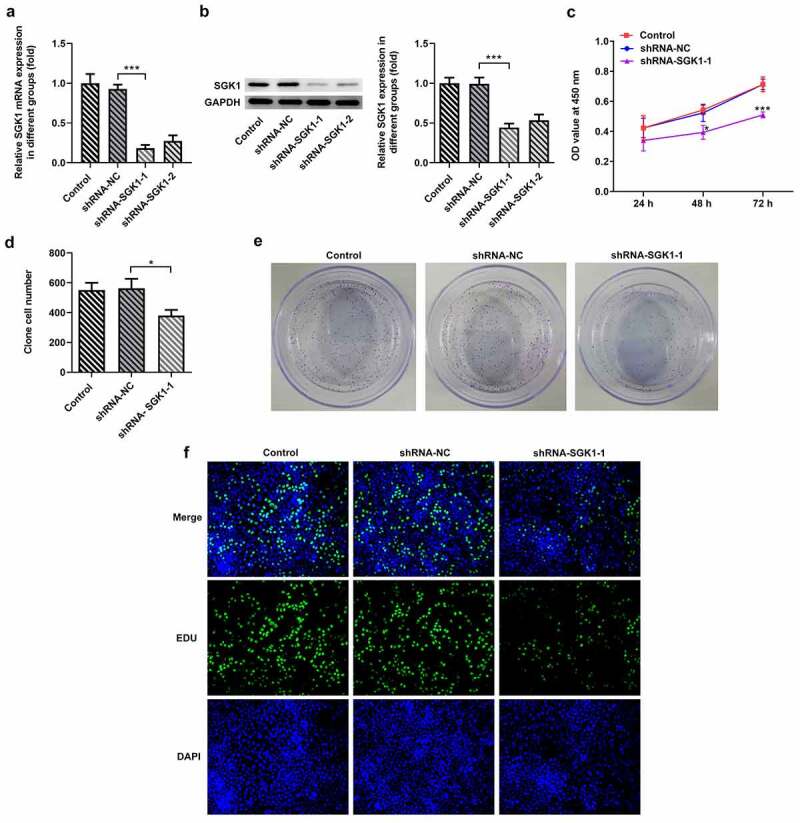
SGK1 silencing suppressed proliferation of KYSE-30 cells. Quantitative PCR (a) and Western blot analysis (b) of SGK1 mRNA and protein, respectively, the detection of cell proliferation through CCK8 assay (c), colony formation and EDU staining (d). The experimental data were represented by mean±SD. Asterisks indicate that difference between two groups is statistically significant. *p < 0.05, ***p < 0.001

From the two designed ShRNA-SGK1-1 and ShRNA-SGK1-2 lentivirus, the one with better interference effect was screened out for further study. The expression levels of SGK1 mRNA and SGK1 protein in KYSE30 cells were determined by real-time PCR and Western blot, respectively. The results showed that the levels of mRNA and SGK1 protein in KYSE30 cells undergoing ShRNA-SGK1-1 transfection were significantly decreased compared to ShRNA-SGK1-2 group, and the difference was statistically significant (Figure 6a). Next, the detection of cell proliferation abilities was performed by CCK8 assay, colony formation assay, and EDU staining. As shown in Figure 6, KYSE30 cells showed lower proliferation after SGK1 silencing, compared with control.

SGK1 silencing suppressed the malignant behaviors of KYSE-30 cells

**Figure 7.**
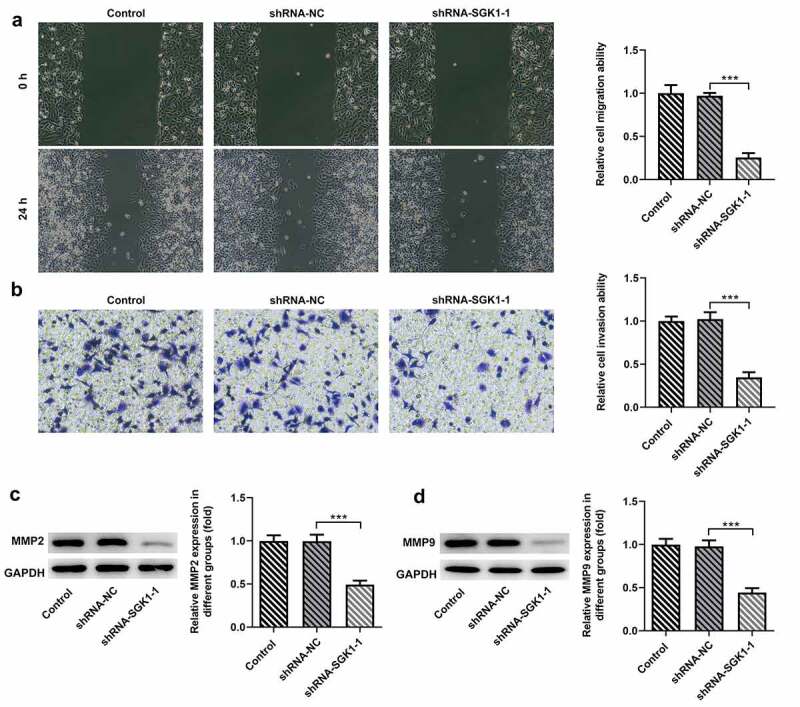
SGK1 silencing inhibited the migration and invasion of KYSE30 cells. The analysis of cell migration through Wound healing assay (a) and invasion through Transwell assay (b), and western blot analysis of MMP2 and MMP9 expression. The experimental data were represented by mean±SD. Asterisks indicate that difference between two groups is statistically significant. ***p < 0.001

Wound healing and transwell assay were used to detect the changes of invasion and migration of KYSE30 cells after ShRNA-SGK1 transfection. The results showed that and the ability of wound healing and the number of transcembrane cells of KYSE30 cells were significantly decreased after ShRNA-SGK1 transfection (Figure 7a, B). Matrix metalloproteinases (MMPS) are highly conserved proteolytic enzymes that can degrade extracellular matrix and destroy the basement membrane of cells, thus playing an important role in the invasion and metastasis of malignant tumors. KYSE30 cells with ShRNA-SGK1 transfection showed lower expression levels of MMP2 and MMP9 than the shRNA-NC group (Figure 7c-D).

SGK1 silencing decreased ESCC tumor growth

**Figure 8.**
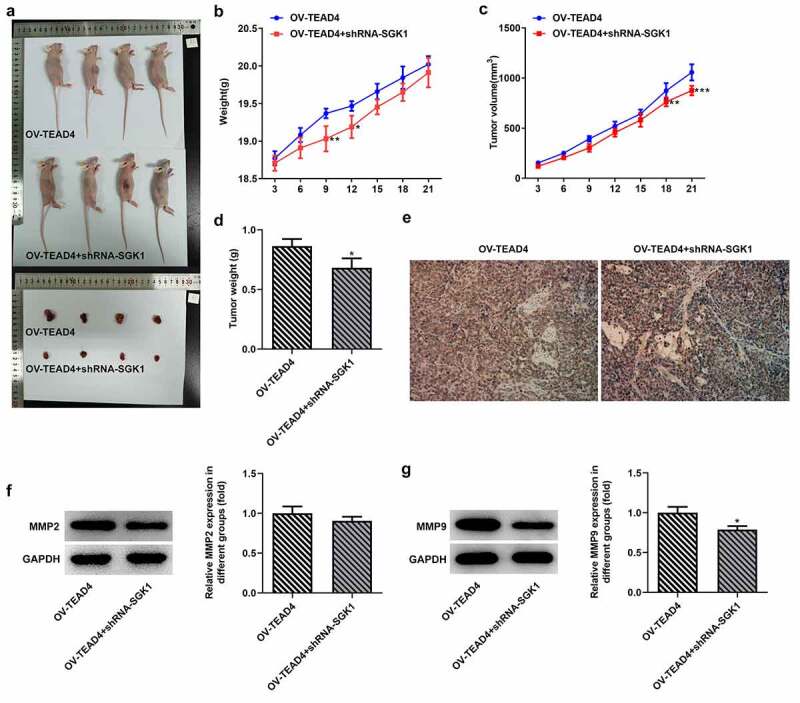
SGK1 could mediate the effect of TEAD4 on tumor growth. The pictures (a) of the weight (b), tumor volume and tumor weight of nude mice. The Ki67 expression (e) through immunostaining assay and the expression of MMP2 and MMP9 (f, g). *p < 0.05, **p < 0.01, ***p < 0.001. Asterisks indicate that difference between two groups is statistically significant. The experimental data were represented by mean±SD

To observe the effect of SGK1 on ESCC tumor growth, KYSE30 cells transfected with OV-TEAD4 or OV-TEAD4+ shRNA-SGK1 were inoculated subcutaneously near the armpit on the right back of nude mice. Mice weight and tumor volume were measured every 3 days from the start of tumor formation in nude mice to observe the growth of transplanted tumors. The subcutaneous tumor was completely exfoliated, and the morphology of the tumor was observed and photographed (Figure 8a). The tumor growth of nude mice in the OV-TEAD4 group was significantly higher than that in the OV-TEAD4+ shRNA-SGK1 group (Figure 8b) on day 9 and day 12 while the tumor volume in the OV-TEAD4 group was higher than OV-TEAD4+ shRNA-SGK1 group on day 18 and day 21. Additionally, tumor weight was markedly increased after SGK1 silencing (Figure 8d). The result of Ki67 immunostaining showed decreased immunostaining and MMP9 expression exhibited the relative low levels in KYSE30 cells with shRNA-SGK1 transfection. These results demonstrated that SGK1 silencing could partly block the effect of TEAD4 on tumor growth.

## Discussion

Esophageal cancer is a common malignant tumor of the digestive tract, and its occurrence and development involve the regulation process of multiple molecules, multiple stages, and multiple factors [13–15]. Although various diagnosis and treatment techniques of esophageal cancer are constantly improved, its 5-year survival rate is extremely low [16,17].

In our work, TEAD4 overexpression was found to significantly upregulate the mRNA and protein levels of YAP1 and CTGF in KYSE-30 cells. A study has reported that the expression of TEAD4 in colorectal adenomas was positively associated with YAP1 expression and regulated its expression through transcription activation [5]. TEAD4 was reported to exhibit upregulation in ESCC compared with adjacent normal esophageal mucosa samples through searching the GEO database [18]. In our work, TEAD4 overexpression significantly increased the proliferation of KYSE-30 cells which was observed by CCK8, colony formation, and EDU staining, as well as the malignant behaviors of KYSE-30 cells including proliferation, migration, and invasion, indicating that TEAD4 could be closely associated with the progression of ESCC. Similarly, TEAD4 was previously investigated to facilitate the malignant behaviors of some cancer cells, such as those in gastric cancer, head neck squamous cell carcinoma, and esophageal cancer [19–21]. A study found that TEAD4 bound to the promoter of LncRNA MNX1-AS1 to induce its transcription, thereby implicating in the progression of gastric cancer [21]. Recently, a research reported YAP1 formed a complex with TEAD4 to affect CCNE1 and CCNE2 expression and cell proliferation [22]. In the present study, there existed an interaction between TEAD4 and YAP1, which was validated through immunoprecipitation assay where anti-TEAD4 antibody was used to pull down the complex of TEAD4-YAP1 complex. In addition, the effects of TEAD4 overexpression on malignant cell behaviors were markedly blocked when we used TED-347 to inhibit the interaction of TEAD4 and YAP1 *in vitro*. Furthermore, the damage of TEAD4-Yap1 interaction through TED-347 significantly inhibited the promoting effects of TEAD4 overexpression on tumor growth *in vivo*.

Next, we further found that TEAD4-YAP1 contributed to increases in protein levels of SGK1 and CTGF. KYSE-30 cells with SGK1 silence showed stronger proliferation abilities and enhanced migration and invasion as compared to cells with ShRNA-NC transfection. Intriguingly, through injecting KYSE-30 cells with OV-TEAD4 transfection or in combination with shRNA-SGK1 transfection into nude mice, we found that SGK1 silencing markedly interfered with the effects of TEAD4 overexpression on tumor volume and the expression of Ki67 and MMPs (MMP2 and MMP9). YAP was found to bind to the enhancer region of SGK1 locating on the SGK1 genomic region 7807 [9]. Similarly, the SGK1 promoter activities were significantly enhanced through TEAD4 overexpression when the SGK1 promoter region and its enhancer region locating in genomic region 7807 were cloned into luciferase reporter. At the same time, TED-347 markedly reversed this influence of the TEAD4-YAP1 complex. Cumulative research revealed that SGK1 was implicated in regulating the mTOR-Foxo3a pathway, MEK/ERK/p53 pathway, and MDM2-dependent p53 degradation, which were closely associated with proliferation and apoptosis [23–25]. Herein, SGK1 mediating the malignant progression depending on TEAD4/YAP exerts aforementioned regulatory roles, which could be related to the regulation for these pathways, which deserves further study.

Conclusion

TEAD4/YAP promoted the malignant process of ESCC cells, which was inhibited by interference with SGK1. The study of the relevant mechanisms affecting the proliferation, migration, and invasion of esophageal cancer cells is expected to improve the prognosis of patients with esophageal cancer and provide new ideas for the treatment of esophageal cancer.

## Highlights

1. The interaction of TEAD4 and YAP was confirmed in the ESCC cell.
2. TEAD4/YAP complex regulated the transcription of SGK1.
3. TEAD4/YAP complex promoted the malignant progression of ESCC.
4. Targeting TEAD4/YAP complex or SGK1 could provide novel sights for developing novel therapies for patients with ESCC.
